# Association between Dietary Intake and Autistic Traits in Japanese Working Adults: Findings from the Eating Habit and Well-Being Study

**DOI:** 10.3390/nu11123010

**Published:** 2019-12-09

**Authors:** Mieko Nakamura, Tomomi Nagahata, Ayako Miura, Eisaku Okada, Yosuke Shibata, Toshiyuki Ojima

**Affiliations:** 1 Department of Community Health and Preventive Medicine, Hamamatsu University School of Medicine, Hamamatsu 431-3192, Japan; eisaku@hama-med.ac.jp (E.O.); shibata@hama-med.ac.jp (Y.S.); ojima@hama-med.ac.jp (T.O.); 2 Department of Nutrition, School of Health and Nutrition, Tokaigakuen University, Nagoya 468-8514, Japan; nagaha-t@tokaigakuen-u.ac.jp; 3 Department of Health and Nutrition, Faculty of Health Proportional Sciences, Tokoha University, Hamamatsu 431-2102, Japan; miura@hm.tokoha-u.ac.jp

**Keywords:** diet, food selectivity, sensory-related behavior, autism

## Abstract

“Autistic traits” include a wide range of severity levels. They are often subclinical, and widely distributed in the general population. It is possible that food selectivity due to hyper- or hypo-reactivity to smell or texture, an autistic feature, may result in inadequate nutrient intakes even among non-clinical adults with autistic traits. However, dietary intake of adults with autistic traits has not been elucidated. This study aimed to investigate an association between dietary intake and autistic traits. We cross-sectionally analyzed data of 1440 men and 613 women extracted from the Eating Habit and Well-Being study. Autistic traits were assessed using the Japanese version of the Subthreshold Autism Trait Questionnaire (SATQ), and dietary intake was assessed using a validated food frequency questionnaire. Iron and vitamin B_12_ intakes were marginally and inversely associated with the SATQ score in men. The SATQ score in women was positively associated with carbohydrate intake, but had an inverse association with protein, mineral, vitamin, and dietary fiber intakes. Low intakes of seaweed, fish and shellfish, and vegetables were observed in participants with severe autistic traits. Associations of autistic traits with food selectivity and low nutrient intakes should be further investigated to promote a new dietary strategy in the general population.

## 1. Introduction

Autism spectrum disorder (ASD) has been accepted as a neurobiological disorder. It is characterized by behavioral diagnosis as social-communication impairments and restricted, repetitive behaviors in the fifth edition of the Diagnostic and Statistical Manual of Mental Health Disorders (DSM-5) [1]. In recent years, several self-administered, quantitative scales have been developed to evaluate “autistic traits” in adults with normal intelligence [2,3,4,5]. Autism lies on a continuum of social-communication disabilities, and autistic traits include a wide range of severity levels. In addition, autistic traits are often subclinical (below the threshold of an ASD diagnosis) [2,3,4,5]. Recent epidemiological studies have revealed that autistic traits are widely distributed in the non-clinical general working population [4,6]. Autistic traits, as well as ASD, are closely associated with mood symptoms and suicidality, and some comorbid mental disorders, such as panic disorder or eating disorders, seem to be more common in patients with autistic traits than in those with ASD [7].

The domain of restricted repetitive behaviors of ASD includes four subdomains: (i) stereotyped or repetitive motor movements, use of objects, or speech; (ii) insistence on sameness, inflexible adherence to routines, or ritualized patterns of either verbal or nonverbal behavior; (iii) highly restricted, fixated interests that are abnormal in intensity or focus; and (iv) hyper- or hypo-reactivity to sensory input or unusual interest in sensory aspects of the environment [1]. The fourth subdomain has been added to DSM-5 for the first time, although behaviors related to hyper- or hypo-reactivity to sound, visual stimuli and light, smell, touch, pain, heat and cold are very common in young children with autism [1,8].

Food selectivity is considered one of such sensory-related behaviors [1,8]. Feeding problems related to picky eating are commonly observed among children with autism, and inadequate nutrient intake due to picky eating has been also reported [9,10,11,12]. It is possible that food selectivity due to hyper- or hypo-reactivity to smell or texture may result in inadequate nutrient intakes even among non-clinical adults with autistic traits. However, little has been known about food selectivity among adolescents and adults with autism [13,14], and food selectivity and nutrient intake of adults with autistic traits has not been elucidated. The current study aimed to investigate an association between dietary intake and autistic traits in Japanese workers.

## 2. Materials and Methods 

### 2.1. Study Design and Participants

This study used data from the Eating Habit and Well-Being (Eat-Well) study of Japanese manufacturing workers conducted between December 2013 and February 2014 [15]. In the Eat-Well study, data on health and lifestyle status were obtained using a self-administered questionnaire, and dietary intake was assessed using a validated food frequency questionnaire (FFQ) [16] with the support from managing units of 43 companies. Further details of the study design and participants were described elsewhere [15]. Informed consent was obtained via submission of the completed questionnaires. The study protocol was approved by the Ethics Committee of the Hamamatsu University School of Medicine (no. 25-203).

### 2.2. Measurement

Autistic traits were assessed using the Japanese version of the Subthreshold Autism Trait Questionnaire (SATQ) [3,4]. The SATQ was a self-administered 24-item questionnaire developed by Kanne et al., which was used to assess a broad range of subthreshold autistic traits in a general population [3]. Its Japanese version was developed by Nishiyama, et al. via a translation-backward translation procedure [4]. Each item of the SATQ was rated on a 0- to 3-point Likert scale; therefore, the total score ranged from 0 to 72 points, with higher scores indicating more severe autistic traits [3]. When using Kanne et al.’s SATQ, the mean total score (standard deviation) in adult participants with ASD was 40.8 (13.6), compared to 23.1 (7.1) in non-ASD controls [3]. Meanwhile, the figures calculated using Nishiyama et al.’s were 45.2 (10.3) and 31.0 (8.0), respectively [4].

Dietary intake was evaluated using the validated 87-food item FFQ developed for Japanese people [16]. The FFQ consists of questions about the usual consumption rates and portion sizes within the previous month. The consumption frequencies include five beverage categories and six food categories. The portion sizes include three food categories, and cup of drinks for beverages. Total energy, nutrient and food intakes were calculated based on the standard tables of food composition in Japan [17]. Energy-adjusted nutrient intake was assessed using the nutrient residual model [18]. Food intake was assessed per 1000 kcal of the total energy intake (g/1000 kcal). Further details of the dietary assessment were described elsewhere [15,16].

### 2.3. Statistical Analyses

In total, 2382 participants were included in the Eat-Well study. Only 2369 people aged 18–79 years had their sex identified, and only 2159 participants had the total energy intake estimated to range between 500 kcal/day and 4000 kcal/day using the FFQ. However, 106 participants with missing SATQ scores were excluded from the analysis. Therefore, the final sample size consisted of 2053 people, including 1440 men and 613 women.

The SATQ scores were categorized into three groups; low (less than 30 points); moderate (equal or higher than 30 points, and less than 40 points); and high (equal or more than 40 points), based on its mean values and the distribution of ASD cases and non-ASD controls in the previous studies [3,4]. In order to obtain crude or age-adjusted standardized β, the association of the SATQ score with the nutrient or food intake was examined using simple or multiple linear regression analyses. In these analyses, each nutrient or food intake was treated as the dependent variable and the SATQ score as an independent variable. The age-adjusted means of nutrient or food intake by sex and the SATQ category were obtained by performing analysis of covariance, and the proportion of food intake by the SATQ category by sex was graphically presented.

The statistical analyses were conducted with IBM SPSS Statistics 25 (IBM, New York, NY, USA). All tests of significance were 2-tailed, and the level of significance was set at *p* < 0.05.

## 3. Results

**Figure 1:**
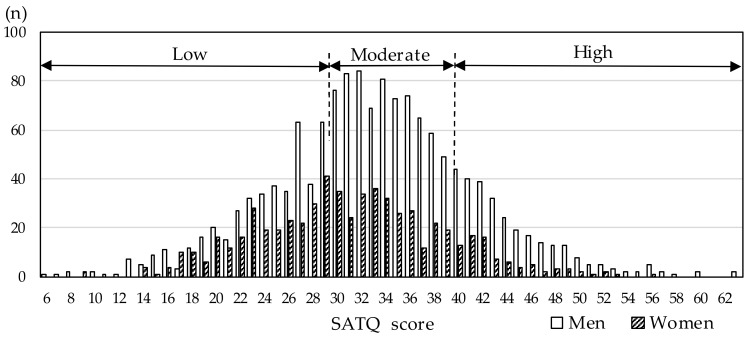
Distribution of the Subthreshold Autism Trait Questionnaire (SATQ) scores by sex. Study participants were categorized into three groups with low, moderate, and high SATQ scores.

The distribution of the SATQ score by sex is shown in Figure 1. The mean (standard deviation) and median (interquartile range) of the SATQ score were 33.2 (8.2) and 33 (28–38) points in men, and 31.0 (7.6) and 31 (26–36) points in women, respectively. Besides, male participants with high and moderate SATQ scores accounted for 20.3% (292/1440) and 49.5% (713/1440), respectively. The corresponding proportions for females were 13.5% (83/613) and 43.6% (267/613).

Table 1 shows the association of the SATQ score with the total energy and nutrient intake. In the male group, iron (*p* = 0.050) and vitamin B_12_ (*p* = 0.051) intakes were marginally and inversely associated the SATQ score, when adjusting for age. In the female group, only carbohydrate intake (*p <* 0.001) was positively associated with the SATQ score, while the intakes of other nutrients, including protein, fat, minerals, vitamins, and dietary fiber, showed inverse associations with the SATQ score. All differences were statistically significant [protein (*p* < 0.001), fat (*p* = 0.020; mono-unsaturated fatty acids, *p* = 0.039; and polyunsaturated fatty acids, *p* = 0.008), minerals (Sodium, *p* = 0.002; Potassium, *p <* 0.001; Calcium, *p* = 0.001; Magnesium, *p <* 0.001; Iron, *p <* 0.001; and Zinc, *p* = 0.036), vitamins (vitamin A, *p <* 0.001; β-carotene, *p* = 0.049; vitamin D, *p* = 0.002; vitamin E, *p <* 0.001; vitamin K, *p* = 0.003; vitamin B_1_, *p* = 0.001; vitamin B_2_, *p <* 0.001; vitamin B_6_, *p <* 0.001; vitamin B_12_, *p* = 0.003; Folic acid, *p <* 0.001; and vitamin C, *p <* 0.001), and dietary fiber (*p* = 0.001)].

Age-adjusted means of nutrient intake by quartile of the SATQ score and sex was obtained by the analysis of covariance, and are shown in Supplemental Tables S1 (men) and S2 (women). In the male group, lower iron, and vitamin B_12_ intakes were associated with the high SATQ score, and lower intakes of protein, magnesium, folic acid, and vitamin C had marginal associations with the high SATQ score. In the female group, high intakes of carbohydrate, and low intakes of dietary fiber, protein, minerals (sodium, potassium, calcium, magnesium, iron), and vitamins (vitamin A, D, E, K, B_1_, B_2_, B_6_, B_12_, C, folic acid) were associated with the high SATQ score.

Table 2 shows the association between food intake and the SATQ score. When adjusting for age, the intake of seaweed (*p* < 0.001) and that of fish and shellfish (*p* = 0.050) were inversely associated with the SATQ score in men. For women, grain product (*p* < 0.001) and sweet (*p* = 0.022) intakes were positively associated with the SATQ score. However, the intakes of vegetables (*p* = 0.007), mushrooms (*p* = 0.055), fruits (*p* = 0.051), soy and soy products (*p* = 0.056), fish and shellfish (*p* = 0.068), and meats (*p* = 0.052) were inversely associated with the SATQ score.

Age-adjusted means of food intake and the SATQ category by sex were obtained by analysis of covariance, and are shown in Supplemental Tables S3 (men) and S4 (women). The low intakes of seaweeds, and fish and shellfish were associated with the high SATQ scores in men. Besides, the high intakes of grain products and low intakes of vegetables were associated with the high SATQ scores in women.

**Figure 2:**
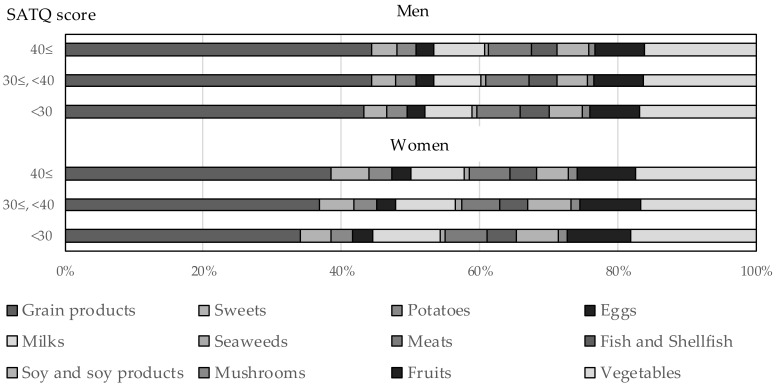
Proportion of food intake by sex and the SATQ category.

Figure 2 presents the proportion of food intake by sex and the SATQ category using age-adjusted means shown in Supplemental Tables S3 and S4. Associations between food intake and the SATQ score were more evident in women than in men.

## 4. Discussion

This cross-sectional study revealed that autistic traits were associated with low intakes of nutrients, including mainly vitamins and minerals, even in non-clinical adults from the general working population. Low consumption of vitamins and minerals may be associated with that of certain foods, such as vegetables, seaweeds, as well as fish and shellfish, based on food selectivity as a characteristic of autism. The proportion of severe autistic traits in men was higher than that in women; however, the association between autistic traits and dietary intake in women was more evident than that in men.

To the best of our knowledge, nutrient intake stratified by sex in adolescents or adults with autism or autistic traits has not been investigated. Indeed, no studies have been directly focused on this matter. Kuschner et al. reported that adolescents and young adults with autism were more likely to prefer familiar foods and dislike foods with particular textures and strong flavors [13]. In addition, Goldschmidt et al. pointed out the importance of providing assistance or training in eating, meal planning and preparation for autistic adults [14].

Nutrient intake among children with autism has been more investigated [9,10,11,12]. A meta-analysis by Sharp et al. revealed that children with autism were five times more likely to experience feeding problems than their peers, and had lower intake of calcium and protein [9]. Liu et al. reported that children with autism consumed fewer macronutrients and had a higher proportion of blood vitamin A deficiency [11]. Malhi et al. pointed that children with autism consumed fewer intakes of foods, particularly fruits and vegetables, accompanied by lower intakes of potassium, copper, and folate than typically developing children [12]. However, sex-stratified analysis was not performed in these studies, as they included mainly boys with autism [9,11,12]. 

In this study, there existed an association between autistic traits and nutrient intake was more evident in the female group than in the male group; however, the reason of this cannot be specified. In the cultural or social contexts, women such as mothers and wives generally plan and prepare meals in Japan. Therefore, many working men with autistic traits may eat balanced meals with a variety of foods planned and prepared by their non-autistic wives. Meanwhile, many working women with autistic traits may have unbalanced meals with selected and limited food varieties that they themselves planned and prepared. Otherwise, in the psychological context, Dell’Osso et al. has reported that patients with eating disorders, including anorexia nervosa and bulimia nervosa, showed severe autism traits [19]. Because eating disorders are more common in women, it is possible that women may be more vulnerable to sensory input of foods compared to men. Why low nutrient intake accompanying with food selectivity is more evident in women with autistic traits than in men with autistic traits should be further investigated in future studies.

This study has several limitations that should be addressed. First, the causal relationship between dietary intake and autistic traits could not be established due to the cross-sectional nature of this study. Second, this study was conducted in a relatively small subset of the population residing in a specific region of Japan. Therefore, this preliminary association between autistic traits and dietary intake in non-clinical adults should be further investigated in other populations. Third, this study did not investigate the history of clinically diagnosed eating disorders, so we cannot exclude them in the present analysis. Forth, people planning and preparing meals were not specified in this study, so we cannot evaluate whether the diet was more influenced by themselves or by the people who plan and prepare the meal.

## 5. Conclusion

The findings of this study provide some insights from the viewpoint of public health nutrition. Diet is one of the key determinants to prevent cancer and cardiovascular disease [20], and recent research has proposed that diet has a preventive effect on mental health [21,22]. Furthermore, a recent cohort study showed that long-term healthy diet quality was associated with brain health indicated by a larger total hippocampal volume [23]. In order to promote physical and mental health, a novel dietary strategy considering food selectivity with autistic traits in the general population should be developed. Moreover, these preliminary results should be further investigated in future studies. More specifically, observational studies should be conducted in other populations, and interventional studies are needed to explore what effects training may have on eating, as well as meal planning and preparation for adults with autistic traits.

## Figures and Tables

**Table 1:** Association between nutrient intake and the SATQ score.

	Men (*n* = 1440)				Women (*n* = 613)			
	Crude		Age-Adjusted		Crude		Age-Adjusted	
	β	*p*-Value	β	*p*-Value	β	*p*-Value	β	*p*-Value
Total energy, kcal	−0.001	0.977	0.001	0.974	−0.061	0.130	−0.058	0.146
Carbohydrate, g	0.040	0.130	0.032	0.218	0.154	< 0.001	0.156	< 0.001
- Dietary fiber, g	−0.040	0.125	−0.030	0.253	−0.131	0.001	−0.125	0.001
Protein, g	−0.036	0.168	−0.028	0.284	−0.163	< 0.001	−0.158	< 0.001
Fat, g	0.018	0.503	0.008	0.764	−0.091	0.024	−0.093	0.020
- SFA, g	0.023	0.391	0.012	0.644	−0.062	0.123	−0.065	0.104
- MUFA, g	0.017	0.507	0.006	0.823	−0.080	0.047	−0.083	0.039
- PUFA, g	0.010	0.701	0.004	0.883	−0.107	0.008	−0.107	0.008
Sodium, mg	−0.024	0.356	−0.017	0.507	−0.129	0.001	−0.124	0.002
Potassium, mg	−0.033	0.216	−0.018	0.486	−0.161	< 0.001	−0.154	< 0.001
Calcium, mg	−0.028	0.294	−0.019	0.478	−0.139	0.001	−0.134	0.001
Magnesium, mg	−0.062	0.018	−0.044	0.084	−0.170	< 0.001	−0.163	< 0.001
Iron, mg	−0.066	0.013	−0.051	0.050	−0.145	< 0.001	−0.139	< 0.001
Zinc, mg	−0.019	0.476	−0.019	0.479	−0.088	0.030	−0.083	0.036
Copper, mg	−0.010	0.706	0.001	0.978	−0.051	0.207	−0.045	0.248
Manganese, mg	−0.027	0.298	−0.008	0.762	−0.041	0.312	−0.034	0.376
Vitamin A, µgRE	−0.024	0.364	−0.015	0.571	−0.149	< 0.001	−0.145	< 0.001
β-carotene, µg	−0.002	0.949	0.002	0.948	−0.082	0.044	−0.079	0.049
Vitamin D, µg	−0.036	0.174	−0.020	0.446	−0.130	0.001	−0.126	0.002
Vitamin E, mg	−0.002	0.954	0.000	0.996	−0.145	< 0.001	−0.143	< 0.001
Vitamin K, µg	−0.054	0.040	−0.047	0.071	−0.122	0.002	−0.120	0.003
Vitamin B_1_, mg	−0.014	0.598	−0.015	0.580	−0.138	0.001	−0.135	0.001
Vitamin B_2_, mg	−0.025	0.347	−0.011	0.676	−0.151	< 0.001	−0.146	< 0.001
Vitamin B_6_, mg	−0.059	0.026	−0.037	0.138	−0.178	< 0.001	−0.170	< 0.001
Vitamin B_12_, µg	−0.065	0.013	−0.051	0.051	−0.121	0.003	−0.117	0.003
Folic acid, µg	−0.060	0.023	−0.040	0.118	−0.158	< 0.001	−0.151	< 0.001
Vitamin C, mg	−0.055	0.038	−0.032	0.207	−0.150	< 0.001	−0.142	< 0.001

Standardized β was obtained by regression analysis with nutrient intake as the dependent variable and the SATQ score as an independent variable. SFA, saturated fatty acids; MUFA, mono-unsaturated fatty acids; PUFA, polyunsaturated fatty acids.

**Table 2:** Associations between food intakes and the SATQ scores.

	Men (*n* = 1440)				Women (*n* = 613)			
	Crude		Age-Adjusted		Crude		Age-Adjusted	
	β	*p*-Value	β	*p*-Value	β	*p*-Value	β	*p*-Value
Grain products	0.026	0.322	0.019	0.467	0.145	< 0.001	0.144	< 0.001
Potatoes	−0.027	0.302	−0.022	0.409	0.035	0.384	0.039	0.327
Vegetables	−0.040	0.129	−0.033	0.209	−0.111	0.006	−0.108	0.007
Mushrooms	−0.052	0.049	−0.046	0.080	−0.080	0.047	−0.077	0.055
Seaweeds	−0.115	< 0.001	−0.108	< 0.001	−0.062	0.128	−0.059	0.143
Fruits	−0.006	0.833	0.008	0.760	−0.082	0.042	−0.076	0.051
Soy and soy products	−0.012	0.637	−0.006	0.806	−0.078	0.054	−0.077	0.056
Fish and Shellfish	−0.069	0.009	−0.050	0.050	−0.077	0.057	−0.071	0.068
Meats	−0.011	0.670	−0.024	0.362	−0.077	0.056	−0.078	0.052
Eggs	0.003	0.898	0.005	0.838	−0.001	0.971	−0.002	0.956
Milks	0.012	0.638	0.017	0.530	−0.047	0.241	−0.046	0.256
Sweets	0.046	0.080	0.035	0.184	0.094	0.019	0.092	0.022

Standardized β was obtained by regression analysis using food intake as the dependent variable and the SATQ score as an independent variable. Unit of food intake: g/1000 kcal.

## Supplementary Materials

The following tables are available online at https://www.mdpi.com/2072-6643/11/12/3010/s1: Table S1: Age-adjusted means of nutrient intake according to quartile of SATQ score in men; Table S2: Age-adjusted means of nutrient intake according to quartile of the SATQ score in women; Table S3: Age-adjusted means of food intake according to quartile of the SATQ score in men; Table S4: Age-adjusted means of food intake according to quartile of the SATQ score in women.
