# Supplementary material for: Association between Dietary Intake and Autistic Traits in Japanese Working Adults: Findings from the Eating Habit and Well-Being Study
Source: Nutrients. 2019 Dec 9;11(12):3010. doi: 10.3390/nu11123010 (PMC6950727; doi:10.3390/nu11123010)
Supplement: Supplementary file 1 [file nutrients-11-03010-s001.pdf]

Supplemental Table S1. Age-adjusted means of nutrient intake according to quartile of SATQ score in men.

	Quartile 1 (0-26)			Quartile 2 (27-31)			Quartile 3 (32-36)			Quartile 4 (37-72)			*p*-value
	Mean	95% CI		Mean	95% CI		Mean	95% CI		Mean	95% CI		
Total energy, kcal	2,029	1,968	2,090	2,019	1,958	2,080	2,012	1,949	2,075	2,005	1,941	2,069	0.956
Carbohydrate, g	278	273	282	282	277	286	283	279	288	281	276	285	0.412
- Dietary fiber, g	11.2	10.9	11.5	11.0	10.7	11.3	11.1	10.8	11.4	10.8	10.5	11.1	0.311
Protein, g	61.7	60.5	62.8	62.1	61.0	63.2	60.5	59.3	61.6	60.4	59.2	61.6	0.090
Fat, g	47.9	46.7	49.2	49.0	47.8	50.3	48.0	46.7	49.3	48.7	47.4	50.0	0.587
- SFA, g	13.9	13.5	14.3	14.2	13.8	14.6	13.9	13.5	14.4	14.2	13.8	14.7	0.579
- MUFA, g	17.2	16.7	17.7	17.7	17.2	18.2	17.3	16.8	17.8	17.5	17.0	18.0	0.497
- PUFA, g	10.9	10.6	11.2	11.1	10.8	11.4	10.8	10.5	11.1	10.9	10.6	11.2	0.634
Sodium, mg	3,485	3,395	3,575	3,476	3,386	3,566	3,409	3,316	3,502	3,411	3,317	3,505	0.513
Potassium, mg	2,101	2,049	2,154	2,085	2,033	2,137	2,050	1,996	2,104	2,057	2,003	2,112	0.505
Calcium, mg	411	395	426	404	388	419	399	383	415	399	382	415	0.702
Magnesium, mg	226	222	231	223	218	228	220	215	225	217	212	222	0.060
Iron, mg	7.24	7.05	7.44	7.16	6.97	7.36	6.95	6.75	7.15	6.89	6.69	7.10	0.042
Zinc, mg	7.68	7.57	7.79	7.73	7.62	7.84	7.62	7.51	7.73	7.58	7.47	7.69	0.250
Copper, mg	1.12	1.11	1.14	1.13	1.11	1.15	1.13	1.11	1.15	1.11	1.09	1.13	0.383
Manganese, mg	3.73	3.62	3.84	3.81	3.70	3.92	3.75	3.64	3.87	3.62	3.50	3.74	0.161
Vitamin A, μgRE	379	364	393	379	365	394	369	354	384	375	360	391	0.763
β-carotene, μg	488	458	517	490	461	520	458	428	489	509	478	540	0.147
Vitamin D, μg	4.98	4.65	5.32	4.95	4.61	5.28	4.61	4.27	4.96	4.66	4.31	5.01	0.302
Vitamin E, mg	5.17	5.02	5.33	5.21	5.06	5.36	5.08	4.92	5.24	5.19	5.03	5.35	0.683
Vitamin K, μg	163	156	171	162	155	170	160	152	167	153	146	161	0.271
Vitamin B_1, mg	0.89	0.87	0.91	0.89	0.87	0.91	0.87	0.85	0.89	0.89	0.86	0.91	0.551
Vitamin B_2, mg	1.06	1.04	1.09	1.08	1.06	1.11	1.06	1.03	1.09	1.05	1.02	1.08	0.360
Vitamin B_6, mg	1.11	1.09	1.14	1.10	1.07	1.13	1.07	1.04	1.10	1.08	1.05	1.11	0.104
Vitamin B_{12}, μg	4.30	4.13	4.48	4.22	4.05	4.39	4.01	3.83	4.19	3.97	3.79	4.15	0.022
Folic acid, μg	291	283	299	291	283	300	287	278	295	277	268	285	0.064
Vitamin C, mg	88	84	92	87	84	91	88	84	92	82	78	86	0.074

CI, Confidence interval; SFA, Saturated fatty acid; MUFA, Mono-unsaturated fatty acids; PUFA, Poly-unsaturated fatty acids; Age adjusted means were obtained by analysis of covariance.

Supplemental Table S2. Age-adjusted means of nutrient intake according to quartile of SATQ score in women.

	Quartile 1 (0-26)			Quartile 2 (27-31)			Quartile 3 (32-36)			Quartile 4 (37-72)			*p*-value
	Mean	95% CI		Mean	95% CI		Mean	95% CI		Mean	95% CI		
Total energy, kcal	1,831	1,754	1,908	1,828	1,746	1,910	1,703	1,622	1,784	1,782	1,696	1,869	0.097
Carbohydrate, g	270	266	274	274	269	278	279	275	283	280	275	285	0.006
- Dietary fiber, g	13.8	13.4	14.2	13.2	12.8	13.7	12.8	12.3	13.3	13.0	12.5	13.5	0.012
Protein, g	69.6	68.3	71.0	68.1	66.6	69.5	66.6	65.2	68.0	65.8	64.2	67.3	0.001
Fat, g	57.6	56.1	59.1	56.8	55.2	58.5	55.2	53.6	56.8	55.8	54.1	57.5	0.149
- SFA, g	17.1	16.6	17.7	17.1	16.5	17.7	16.4	15.9	17.0	16.6	16.0	17.3	0.249
- MUFA, g	20.4	19.8	21.0	20.1	19.5	20.7	19.5	18.9	20.2	19.9	19.2	20.5	0.211
- PUFA, g	12.7	12.4	13.1	12.4	12.0	12.7	12.3	11.9	12.6	12.2	11.8	12.6	0.208
Sodium, mg	4,009	3,890	4,128	3,892	3,766	4,018	3,793	3,668	3,917	3,772	3,639	3,906	0.031
Potassium, mg	2,581	2,506	2,657	2,477	2,397	2,557	2,377	2,298	2,456	2,377	2,293	2,462	<0.001
Calcium, mg	540	518	563	518	494	541	493	470	517	483	458	508	0.003
Magnesium, mg	266	259	273	256	248	263	250	242	257	245	237	253	0.001
Iron, mg	9.01	8.70	9.31	8.54	8.22	8.87	8.48	8.16	8.79	8.24	7.90	8.58	0.008
Zinc, mg	8.28	8.16	8.41	8.19	8.06	8.32	8.17	8.04	8.30	8.08	7.94	8.22	0.193
Copper, mg	1.23	1.21	1.25	1.21	1.19	1.24	1.22	1.20	1.25	1.20	1.17	1.23	0.401
Manganese, mg	4.14	3.96	4.32	4.03	3.84	4.22	4.06	3.87	4.25	3.89	3.69	4.09	0.333
Vitamin A, μgRE	516	495	537	477	455	499	456	434	477	466	442	489	<0.001
β-carotene, μg	595	556	634	547	506	588	525	484	566	544	500	587	0.088
Vitamin D, μg	6.86	6.35	7.38	6.06	5.52	6.61	5.76	5.22	6.30	5.53	4.96	6.11	0.004
Vitamin E, mg	6.55	6.33	6.77	6.23	6.00	6.47	5.97	5.74	6.20	6.12	5.88	6.37	0.004
Vitamin K, μg	210	199	222	190	178	202	187	175	199	186	173	198	0.012
Vitamin B_1, mg	1.02	0.99	1.04	1.00	0.98	1.03	0.97	0.95	1.00	0.97	0.94	1.00	0.015
Vitamin B_2, mg	1.33	1.29	1.37	1.28	1.24	1.33	1.23	1.19	1.27	1.21	1.17	1.26	0.001
Vitamin B_6, mg	1.26	1.22	1.29	1.23	1.19	1.27	1.17	1.14	1.21	1.15	1.11	1.19	<0.001
Vitamin B_{12}, μg	5.00	4.75	5.24	4.75	4.49	5.01	4.61	4.36	4.87	4.41	4.14	4.69	0.017
Folic acid, μg	375	361	389	354	339	369	338	323	353	336	320	351	<0.001
Vitamin C, mg	123	117	129	117	110	123	107	101	114	108	102	115	0.001

CI, Confidence interval; SFA, Saturated fatty acid; MUFA, Mono-unsaturated fatty acids; PUFA, Poly-unsaturated fatty acids; Age adjusted means were obtained by analysis of covariance.

Supplemental Table S3. Age-adjusted means of food intake according to quartile of SATQ score in men.

	Quartile 1 (0-28)			Quartile 2 (29-33)			Quartile 3 (34-38)			Quartile 4 (39-72)			*p*-value
	Mean	95% CI		Mean	95% CI		Mean	95% CI		Mean	95% CI		
Grain products	262.7	255.3	270.1	266.9	259.5	274.3	270.5	262.9	278.1	264.9	257.1	272.6	0.526
Potatoes	17.4	16.5	18.2	17.5	16.7	18.4	17.4	16.5	18.3	16.7	15.8	17.6	0.604
Vegetables	101.1	96.4	105.8	101.4	96.7	106.0	94.4	89.6	99.2	99.1	94.2	104.0	0.154
Mushrooms	6.8	6.5	7.1	6.6	6.2	6.9	6.4	6.0	6.7	6.2	5.8	6.5	0.075
Seaweeds	4.6	4.3	5.0	3.8	3.5	4.2	3.6	3.2	3.9	3.6	3.2	3.9	<0.001
Fruits	44.5	40.5	48.4	41.7	37.8	45.7	44.5	40.5	48.6	42.6	38.4	46.7	0.699
Soy and soy products	29.0	26.3	31.6	27.1	24.5	29.8	26.1	23.4	28.9	27.4	24.6	30.1	0.523
Fish and Shellfish	25.4	24.0	26.8	24.9	23.5	26.3	22.8	21.3	24.2	22.6	21.1	24.1	0.009
Meats	38.7	37.2	40.3	39.4	37.8	40.9	37.3	35.7	38.9	38.1	36.4	39.7	0.296
Eggs	15.4	14.5	16.3	16.2	15.3	17.1	15.6	14.7	16.5	15.2	14.3	16.2	0.526
Milks	40.2	34.7	45.7	41.3	35.8	46.8	41.0	35.3	46.7	43.7	37.9	49.4	0.856
Sweets	19.8	18.1	21.5	22.2	20.6	23.9	21.3	19.6	23.0	22.2	20.5	24.0	0.148

g/1,000kcal; CI, Confidence interval; Age adjusted means were obtained by analysis of covariance.

Supplemental Table S4. Age-adjusted means of food intake according to quartile of SATQ score in women.

	Quartile 1 (0-26)			Quartile 2 (27-31)			Quartile 3 (32-36)			Quartile 4 (37-72)			*p*-value
	Mean	95% CI		Mean	95% CI		Mean	95% CI		Mean	95% CI		
Grain products	228.7	218.7	238.7	231.0	220.4	241.5	251.4	241.0	261.8	245.8	234.7	257.0	0.004
Potatoes	20.4	19.0	21.8	21.8	20.3	23.3	20.8	19.4	22.3	21.9	20.3	23.5	0.424
Vegetables	126.4	118.9	133.8	114.8	106.9	122.7	108.6	100.8	116.4	112.5	104.2	120.8	0.009
Mushrooms	9.2	8.6	9.8	8.8	8.1	9.4	8.6	7.9	9.3	8.3	7.6	9.0	0.300
Seaweeds	5.3	4.7	5.9	4.8	4.1	5.5	5.0	4.4	5.7	4.5	3.8	5.1	0.336
Fruits	61.7	54.9	68.4	63.8	56.6	70.9	57.0	49.9	64.1	54.6	47.1	62.2	0.280
Soy and soy products	42.0	36.5	47.6	39.6	33.7	45.6	40.6	34.7	46.4	36.0	29.7	42.2	0.542
Fish and Shellfish	28.8	26.7	31.0	28.2	25.9	30.5	27.0	24.8	29.3	25.2	22.8	27.6	0.145
Meats	40.1	37.8	42.3	39.7	37.3	42.1	36.4	34.0	38.7	38.3	35.8	40.9	0.115
Eggs	18.4	16.8	20.1	19.6	17.8	21.4	19.2	17.5	21.0	19.6	17.7	21.5	0.748
Milks	65.9	55.9	75.8	64.0	53.5	74.6	54.1	43.7	64.5	55.4	44.3	66.5	0.282
Sweets	29.9	26.7	33.1	31.9	28.6	35.3	33.3	30.0	36.6	35.7	32.2	39.3	0.105

g/1,000kcal; CI, Confidence interval; Age adjusted means were obtained by analysis of covariance.
